# Editorial for the Special Issue on Recent Advances in Thin Film Electronic Devices

**DOI:** 10.3390/mi13091445

**Published:** 2022-09-01

**Authors:** Chengyuan Dong

**Affiliations:** Department of Electronic Engineering, Shanghai Jiao Tong University, Shanghai 200240, China; cydong@sjtu.edu.cn

Thin film electronic devices have been attracting more and more attention because of their applications in many industry fields, such as in flat panel displays (FPDs), energy devices, sensors, memories, and so on [1,2,3]. From a fabrication point of view, thin film electronic devices can not only be prepared on rigid substrates (including glass, wafers, etc.) but also on flexible substrates (including polymers, paper, etc.); this makes give thin film electronic devices the potential to be used in some quickly advancing fields, such as the Internet of Things and medical electronics [4,5].

The family of thin film electronic devices includes thin film transistors (TFTs), thin film solar cells (TFSCs), thin film sensors (TFSs), thin film memories (TFMs), and many other conventional and novel devices. To fabricate them, many advanced preparation methods, including magnetron sputtering, chemical vapor deposition (CVD), atomic layer deposition (ALD), lithography, dry etching, as well as solution methods, are employed. To improve the yield for mass productions, some effective testing methods are widely used.

This Special Issue comprises 11 original papers about recent advances in the research and development of thin film electronic devices. Specifically, three research fields are covered: device fundamentals (five papers), fabrication processes (five papers), and testing methods (one paper). These typical studies reveal the recent advances in thin film electronic devices, which are briefly summarized as follows.

Defect density dominates the electrical properties of semiconductor films and devices. In this Special Issue, J. C. Tinoco et al. investigated the impact of the semiconductor defect density on solution-processed flexible Schottky barrier diodes (SBDs) [6]. The simulation analysis and experimental measurements confirmed that it was necessary to consider the presence of a density of states in the semiconductor gap for standard SBDs to understand specific changes observed in their performance.

Nitrogen-doping is an effective method to improve the electrical performance and stability of amorphous InGaZnO (a-IGZO) TFTs. A technology computer-aided design (TCAD) simulation was employed to analyze the nitrogen-doping effect on sub-gap density of states in a-IGZO TFTs [7]. The numerical simulation results displayed that the interface trap states, bulk tail states, and deep-level sub-gap defect states originating from oxygen-vacancy-related defects might be effectively suppressed by an appropriate nitrogen-doping treatment.

In addition to TFTs, the gate-all-around field-effect transistors (GAA FETs) were also simulated to find the mechanisms about reducing power with punch-through current annealing [8]. To maximize power efficiency during electro-thermal annealing, the application of gate module engineering was confirmed to be more suitable than the isolation or source drain modules.

It is interesting that some novel functions could be realized by CsPbI_3_ thin films. J. Y. Chen et al. confirmed the learning and memory behavior similar to biological neurons in Au/CsPbI_3_/ITO structure [9]; they also discussed the possibility of forming long-term memory in the device through changing input signals.

In recent years, many researchers have proposed novel photonic crystal fibers (PCF)-based polarization filters. Here, S. Selvendran et al. [10] used D-shaped PCF to form a reconfigurable surface-plasmon-based filter/sensor; they achieved a maximum confinement loss of about 713 dB/cm at the operating wavelength of 1.98 μm in X-polarization by the surface plasmon effect.

Generally, electrical properties of thin films are influenced by many processing conditions. The effect of the deposition time on the structural and 3D vertical growth and electrical conductivity properties of electrodeposited anatase-rutile nanostructured thin films was studied [11], proving that the deposition time during the electrophoretic experiment consistently evidently affected the structure, morphology, and electrical conductivity of the corresponding films.

Recently, fabrication improvement in metal oxide TFTs has become a hot topic. Two papers related to this issue are included in this Special Issue [12,13]. N. Chen et al. [12] tried to apply laser treatment in the solution-processing of active layers of metal oxide TFTs, covering laser photochemical cracking of metastable bonds, laser thermal effect, photoactivation effect, and laser sintering of nanoparticles. In addition, W. Zhang et al. [13] investigated atmosphere effect in post-annealing treatments for a-IGZO TFTs with SiO_x_ passivation layers, where different atmospheres (air, N_2_, O_2_, and vacuum) were studied at length.

Interestingly, some novel processes relating thin film electronic devices were reported in this Special Issue [14,15]. K. S. Lee employed a process simplification for n-type nanosheet FETs without a ground plane region [14]; the proposed flow could be performed in situ, without the requirement of changing chambers or a high-temperature annealing process. In addition, X. Ding et al. successfully realized the efficient multi-material structured thin film transfer to elastomers for stretchable electronic devices by combining bench-top thin film structuring with solvent-assisted lift-off methods [15].

Liquid crystal displays (LCDs) are still the mainstream of FPDs, so it is very important to use effective inspection methods to improve yield in the mass productions of TFT-LCDs. Accordingly, non-contact optical detection of foreign materials adhered to color filters (CFs) and TFTs was investigated by F. M. Tzu et al. [16]. In contrast to the height of the debris material, the image was acquired by transforming the geometric shape from a square for side-view illumination using area charge-coupled devices (CCDs) in this study. The relating experiments presented a successful design to prevent a valuable component malfunction.

I would like to thank all the authors for submitting their papers to the Special Issue “Recent Advances in Thin Film Electronic Devices”, as well as all the reviewers and editors for their contributions to improving these submissions.
